# Kinetic studies and CFD-based reaction modeling for insights into the scalability of ADC conjugation reactions

**DOI:** 10.3389/fbioe.2023.1123842

**Published:** 2023-04-03

**Authors:** Jan Tobias Weggen, Janik Seidel, Ryan Bean, Michaela Wendeler, Jürgen Hubbuch

**Affiliations:** ^1^ Institute of Process Engineering in Life Sciences, Section IV: Biomolecular Separation Engineering, Karlsruhe Institute of Technology (KIT), Karlsruhe, Germany; ^2^ Purification Process Sciences, BioPharmaceuticals Development, R&D, AstraZeneca, Gaithersburg, MD, United States

**Keywords:** antibody-drug conjugate (ADC), conjugation reaction, computational fluid dynamics (CFD), mixing, scale-up, single-use, kinetic modeling

## Abstract

The manufacturing of antibody-drug conjugates (ADCs) involves the addition of a cytotoxic small-molecule linker-drug (= payload) to a solution of functionalized antibodies. For the development of robust conjugation processes, initially small-scale reaction tubes are used which requires a lot of manual handling. Scale-up to larger reaction vessels is often knowledge-driven and scale-comparability is solely assessed based on final product quality which does not account for the dynamics of the reaction. In addition, information about the influence of process parameters, such as stirrer speed, temperature, or payload addition rates, is limited due to high material costs. Given these limitations, there is a need for a modeling-based approach to investigate conjugation scale-up. In this work, both experimental kinetic studies and computational fluid dynamics (CFD) conjugation simulations were performed to understand the influence of scale and mixing parameters. In the experimental part, conjugation kinetics in small-scale reaction tubes with different mixing types were investigated for two ADC systems and compared to larger bench-scale reactions. It was demonstrated that more robust kinetics can be achieved through internal stirrer mixing instead of external mixing devices, such as orbital shakers. In the simulation part, 3D-reactor models were created by coupling CFD-models for three large-scale reaction vessels with a kinetic model for a site-specific conjugation reaction. This enabled to study the kinetics in different vessels, as well as the effect of process parameter variations *in silico*. Overall, it was found that for this conjugation type sufficient mixing can be achieved at all scales and the studied parameters cause only deviations during the payload addition period. An additional time-scale analysis demonstrated to aid the assessment of mixing effects during ADC process scale-up when mixing times and kinetic rates are known. In summary, this work highlights the benefit of kinetic models for enhanced conjugation process understanding without the need for large-scale experiments.

## 1 Introduction

Antibody-drug conjugates (ADCs) are highly potent biopharmaceuticals that combine the targeting specificity of a monoclonal antibody with the potent cytotoxicity of chemotherapy. In the last decade, ADCs have made considerable progress: In 2021, ten ADCs were approved by the Food and Drug Administration (FDA) and >80 are in clinical trials (Dean et al., 2021). For the coupling of the cytotoxic drug/payload to the monoclonal antibody (mAb), functional groups such as lysine residues or free thiols after reduction of interchain disulfide bonds are used (Tsuchikama and An, 2018). However, these conjugation techniques often cause heterogeneous drug-load profiles and a variety of positional isomers (Matsuda and Mendelsohn, 2021), (Kommineni et al., 2020). In the development of scalable and robust ADC processes, a major challenge is the characterization of critical process parameters (CPP) in each synthesis step that impact critical quality attributes (CQAs) (Matsuda and Mendelsohn, 2021). Especially, the drug-to-antibody ratio (DAR), drug load profile and aggregate level are important CQAs as they directly influence the product safety, efficacy and pharmacokinetics (Stump and Steinmann, 2013). Different site-directed conjugation methods have been developed that aim to synthesize more homogenous ADC products, control the site of attachment, and achieve more stable conjugates (Panowski et al., 2014), (Jackson, 2016)^.^ But even for these methods, product-related impurities, such as under- and over-conjugated species or aggregation still occur (Hutchinson et al., 2018; Cao et al., 2019; Coumans et al., 2020).

At the same time, regulatory agencies increasingly promote the understanding of both product and process already in the development phase according to the concept of *Quality by Design (QbD)* (ICH, 2008). As the needed intermediates for ADCs are costly and difficult to handle, scale-down models (SDM) are often used in process development. These models are typically designed by selecting one scale-down parameter to be similar along scales. However, this approach becomes difficult for larger scale differences, because certain factors, such as the power input per volume ratio (P/V), are impractical to be kept constant. (Marques et al., 2010; Tajsoleiman et al., 2019; Montes‐Serrano et al., 2021). For ADCs, no systematic approach has been reported and scale-comparability is assessed based on constant ADC quality attributes, such as the DAR or aggregate level (Hutchinson et al., 2018).

Due to the ongoing digitalization of bioprocesses, the use of process models describing complex biopharmaceutical processes are promoted (Gargalo et al., 2020). Different types of statistical or computational approaches, such design of experiment (DoE), mechanistic or hybrid models and computational fluid dynamics (CFD) were recently applied, also aiming to extrapolate beyond the design space and predict larger scales (Roush et al., 2020; Smiatek et al., 2020; Sinner et al., 2021). Within the last years, computational fluid dynamics (CFD) have gained more attention for (bio)-reactor scale-up due to the ability to provide high resolution results of the complete flow pattern at various scales (Scully et al., 2020), (Werner et al., 2014). The goal is to establish an advanced process model that allows to examine the effects of scale, turbulence, and mixing parameters completely *in silico*. In the field of biotechnology, CFD was recently applied to study bioreactor mixing performance (Wutz et al., 2020; Xing et al., 2020; Martinetz et al., 2021), predict large-scale mixing times and oxygen mass transfer (Scully et al., 2020), (Bach et al., 2017; Wutz et al., 2018; Nadal-Rey et al., 2022) and explore inhomogeneity effects on the cell metabolism (Haringa et al., 2017). However, most studies focus on comparably slow bio-chemical processes, such as fermentation, with characteristic times in the range of min to hours, while typical chemical reactions being significantly faster (down to nano-sec). There are only a few cases, where CFD and mechanistic models were coupled to predict the mixing effect on the course of chemical reactions (Vicum et al., 2004). Due to high computational demand elegant ways to minimize the computational effort by using compartment modeling (Yang et al., 2019), (Öner et al., 2018) or surrogate models (Kaya et al., 2022) were also developed.

In the field of ADCs, mostly statistical approaches, such as design of experiments (DoE) are utilized to optimize the process and gain a solid understanding of the CPP-CQA relationship (Stump and Steinmann, 2013). For scale-up prediction, the use of process models has not been reported yet. Especially the conjugation step is considered to demand adequate mixing and careful considerations of the payload addition method or rate. In addition, single-use reactors are becoming more commonly used in ADC manufacturing (Marcq and Damelin, 2018), (Schmidhalter et al., 2019). However, their reactor design and mixing geometry is different from conventional stirred vessels which might add further possibilities to affect the product. In an earlier study (Hu et al., 2017), CFD models were generated to compare different reaction vessels based on mixing times simulations, while the importance for ADC reactions was not discussed. Recently, a mechanistic kinetic model for a site-directed conjugation was developed by Andris et al. (Andris et al., 2019), showing that the model could successfully optimize concentrations and reaction times. Since the dataset was limited to small-scale experiments and not compared to large-scale data, evidence of the potential to predict scale-up parameter is still lacking. In addition to the differences in size and geometry, manual handling in small scales vs a higher degree of automation in larger scales may result in fundamental differences in flow characteristics and mass transfer. These effects might impact the conjugation reaction.

Here, we present a thorough investigation of scale effects and mixing parameters on the course of ADC conjugation reactions by applying experimental kinetic studies and the coupling of CFD models with an ADC conjugation kinetic model. In the experimental kinetic studies, the influence of mixing in typically used small reaction tubes is analyzed for two model ADCs aiming to produce robust conjugation kinetics. One ADC has a target DAR value of 2, and conjugation is achieved by site-specific attachment to inserted cysteine residues. The second ADC has a target DAR value of 8, and the conjugation workflow is based on stochastic conjugation to interchain disulfide bonds after reduction. It is shown, how well this test tube scale (∼1 mL) mimics the conjugation reaction in a glass reactor at lab-scale (100 mL) at industrial-relevant concentrations. In the second modeling part, CFD simulations are performed for three differently sized vessels typically used for pilot and large-scale ADC manufacturing (up to 50 L), namely, two conventional glass stirred vessels and one single-use vessel. The steady-state results and mixing time simulations are considered for a CFD-based vessel comparison. Subsequently, a kinetic model for the site-directed conjugation reaction is incorporated in the CFD models resulting in a full 3D reactor model and is further used to study *in silico* how scale and process parameters affect the course of the conjugation reaction.

## 2 Materials and methods

### 2.1 Experimental conjugation kinetic studies with two model ADCs

Two types of kinetic studies with ADC1 and 2 were conducted: 1) Mixing kinetic studies to determine optimal mixing conditions for small-scale conjugation reactions by using either external mixing or internal mixing. 2) Conjugation kinetics with the optimized small-scale conditions vs lab-scale conjugation to evaluate the scale comparability.

#### 2.1.1 Chemicals, ADCs and functionalization steps

Two ADCs were investigated within the experimental part of this study: ADC1 with two engineered cysteines for a site-directed DAR 2 conjugation and ADC2 for a cysteine-linked DAR 8 conjugation. For DAR 2, a functionalized mAb solution was generated through a full reduction with tris(2-carboxyethyl) phosphine hydrochloride (TCEP, EMD Millipore), followed by a buffer exchange using Vivaspin 20 (30 kDa MWCO, Cytiva) and a re-oxidation of the interchain disulfides with (L)-dehydroascorbic acid (DHAA, Sigma-Aldrich). For the ADC2 with a DAR of 8, a mild reduction of the interchain disulfides with TCEP was performed. In both cases, conjugation was carried out with a maleimide-functionalized payload that was dissolved in DMSO (Sigma-Aldrich). All other solutions were prepared with 20 mM sodium phosphate buffer (J.T. Baker), 1 mM EDTA (EMD Millipore), pH 7.0.

#### 2.1.2 Conjugation kinetics

For all studies, functionalized mAb solution was prepared by the procedure described above. MAb solutions with ADC1 were diluted to a concentration of 10 mg/mL and conjugated with 5x molar payload excess. In case of ADC2, a lower concentration of 1.5 mg/mL was tested, and conjugations were performed with 11x molar payload excess. As mixing vessel for the small-scale experiments, reaction tubes (1.5 mL Safe-Lock tubes, Eppendorf) were used. Preliminary studies with ADC2 showed that thorough initial payload mixing is required to prevent lower DAR values or inconsistent kinetics, especially when an orbital shaker is used for subsequent mixing (Supplementary Figure S1). To solely assess the influence of the final mixing, the initial payload mixing was conducted by one end-over-end rotation of the tube (tube rotator, VWR). Final mixing was either achieved by “external” mixing (Eppendorf thermomixer C) or internally with a magnetic stir bar (Magnetic stir bar, Merck, Part #23226) placed inside the tube. Two shaking/mixing speeds for each condition were tested. Conjugations with ADC2 were performed in duplicates.

Lab-scale conjugations were performed to compare the kinetics with the optimized small-scale mixing system. The mAb concentrations were set to 5 mg/mL (ADC1) or to 20 mg/mL (ADC2) and the same payload excesses as in the small-scale were used. The stirrer-based mixing was found to lead to more ideal kinetics in the small-scale reactions and was therefore used for the comparison to the lab-scale kinetics. Lab-scale conjugations were conducted with 100 mL mAb solution in a stirred glass reactor (Chemglass, inner diameter = 108 mm, Model CG-1949-x-300), that was also included in the CFD study (later referred to as GST-1). The anchor stirrer (Chemglass, impeller diameter = 81 mm, Model CG-2081-A-04) was installed so that the stirrer was close to the bottom surface. The stirrer speed was set to 60 rpm and payload solution was manually added with a pipette to the stirred mAb solution.

#### 2.1.3 Reference analytics

To obtain data on conjugation kinetics, samples were taken at defined timepoints over 1 h and immediately quenched with N-Acetyl cysteine (Sigma-Aldrich). Each sample was further treated with reducing buffer, incubated at 37 °C for 30 min and analyzed using reversed-phase ultra-high-performance liquid chromatography (RP-UHPLC). A detailed description of the applied protocol, method and chromatography system can be found in (Cao et al., 2019). The DAR was calculated based on the peak areas of unconjugated/conjugated light and heavy chain peaks.

### 2.2 CFD simulations for large-scale vessels

Multiple CFD simulations were performed, and their individual purpose is shortly described in the following. First, steady-state and transient mixing time simulations were conducted to characterize three industrially relevant mixing vessels. Validation of the CFD models was done based on available mixing times. Next, a calibrated kinetic model for the site-directed conjugation reaction was incorporated in the existing CFD models. This enabled to study the direct impact of mixing geometry and scale on the conjugation kinetic and is referred to as 3D-model. The significance of the 3D-model to accurately describe the conjugation reactions was estimated by comparing the predicted kinetics with the 0D-model (ideal mixing assumption). For one of the studied vessels the influence of varying process parameters was further exemplarily studied. Due to GMP limitations, a validation of the large-scale conjugation kinetic could only be performed for the smallest mixing vessel.

#### 2.2.1 Geometries and meshes

The studied geometries comprise three disparate, unbaffled vessels: A 300 mL “lab-scale” glassed stirred tank (GST-1) equipped with an anchor stirrer (Chemglass, Model CG-1949-x-300), a 50 L “large-scale” glass stirred tank (GST-2) equipped with a 45 pitched-blade stirrer (Chemglass, Model CG-1968-81) and a 50 L “large-scale” single-use mixer (SUM) equipped with an eccentric bottom-mounted agitator (Mobius MIX Bag, Merck Millipore). Liquid volumes and stirrer speeds were selected to be comparable to real process conditions. All geometries were designed in ANSYS DesignModeler. An overview of the vessels and the parameters is given in Table 1. The exact dimensions are tabularized in the Supplementary S2. For the GST-1, the whole volume was modelled as a single rotating frame having the same rotational speed as the stirrer. For both large-scale vessels (GST-2 and SUM), the fluid domain was divided into two zones, a cylindrical rotating zone around the impeller and a stationary zone for the remaining volume, to model the stirrer motion using the multiple reference frames (MRF) approach. The water surface was assumed to be flat. The geometries were discretized with Poly-Hexcore meshes using the integrated FLUENT mesher. Five boundary layers of prism cells were applied for the walls (vessel, impeller and shaft) in order to resolve the transition of the flow in the near-wall region. The mesh close to the impeller was further refined because of higher expected gradients. The resulting meshes consisted of approximately 687,000 (GST-1), 26,000 (GST-2) and 38,000 (SUM) mesh elements per liter. The higher mesh cell density for the GST-1 was due to the larger impeller area relative to the volume which had to be refined. Overall, a minimum orthogonality of 0.2 or greater and a maximum skewness less than 0.8 was achieved. To judge sufficient spatial discretization, mesh independency tests were performed for each vessel at the highest stirrer speed based on global average velocity magnitude and turbulence parameters (Supplementary Figure S4). The final meshes and mesh metrics are depicted in the Supplementary S3.

**TABLE 1:** Geometries and parameters of the studied reaction vessels. *For GST-2, three stirrer speeds were investigated in the parameter study. **For the SUM a lower stirrer speed of 250 rpm was added because mixing time data were available only for this stirrer speed.

3D view	Name	Volume liquid	Impeller type	Speed/rpm	Re_imp_
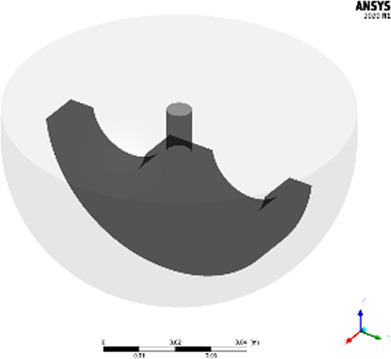	Lab-scale glass stirred tank **(GST-1)**	300 mL	2 blades, anchor-style, centric	60	6,218
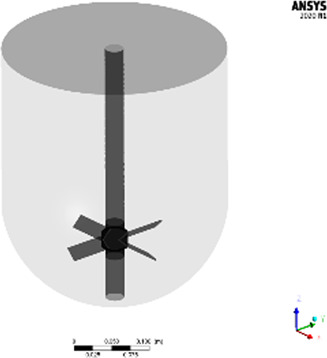	Large-scale glass stirred tank **(GST-2)**	22 L	4 blades, pitched bladed (45°), centric	60, 80*, 120*	22141,29,522, 44,282
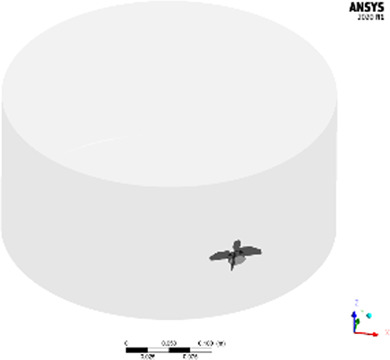	Single-use mixer **(SUM)**	25 L	4 blades, 15° angle, bottom mounted, eccentric	250**, 400	15,765, 25,224

#### 2.2.2 Steady-state simulations and P/V ratio

Steady-state CFD simulations were performed for the predictions of the stationary flow field. All simulations were run using the finite volume method with pressure-based solvers in ANSYS Fluent v2020 R2. Turbulence models are required since all impeller Reynolds numbers (Re_imp_) are in the transitional or fully turbulent regime. In a preliminary study, two frequently used Reynolds-averaged Navier Stokes (RANS) models, namely, the k-ε-RNG and the Reynolds Stress Model (RSM) model, were compared. Since the k-ε-RNG was found to be more stable and achieve similar results in less computational time, it was applied in this work. The physical properties of the fluid were assumed to be equal to water with 10% DMSO (a typical media composition for conjugation reactions). The density was set to 1,010.5 kg m^-3^ and the dynamic viscosity to 0.00106 kg m^-1^ s^-1^. The liquid surface was defined as no-shear (free slip), tank and stirrer walls were treated with zero velocity (no-slip) boundary condition. The near-wall region was modeled with standard wall function. The SIMPLEC algorithm was used for pressure-velocity coupling. Further, the second-order upwind scheme for interpolation and Green-Gause node based for gradient determination were used. The simulations were run for at least 10,000 iterations and convergence was judged based on continuity of volume-averaged velocity magnitude, impeller torque and turbulent energy dissipation ε) as well as scaled residuals. The flow was assumed to be stationary when no considerable deviation of these values was observed (data are shown in the Supplementary Figure S5). The stationary impeller torque (*M*) was used to calculate the simulated *P/V*:

$$\begin{array}{c} \frac{P}{V}=\frac{2\pi *N*M}{V} \end{array}$$ (1)

where *N* is the stirrer speed and *V* is the liquid volume.

#### 2.2.3 Mixing time studies—Computational

Tracer simulations using the species transport model were performed for computational mixing time studies. The flow in each vessel was initialized with the respective steady-state result and subsequently “frozen”. This approach was expected to be valid, since the flow fields in GST-1 and GST-2 were considerably steady and did not fluctuate (see Supplementary Figure S5). For the SUM, the flow field fluctuated slightly, but a comparison between frozen and dynamic approach showed only minor differences in the simulated mixing times which justifies the use of the frozen approach also in this case. After the initialization, a non-reactive species mimicking a 1 M NaCl solution was added below the water surface at a position analogue to experimental procedure (exact addition positions are described in Supplementary S3). With the defined tracer volume, a tracer concentration of 0.1% (v/v) was reached. The tracer diffusion coefficient was specified to D = 1*10^−9^ m^2^/s. A first-order implicit methods for the temporal discretization was used. Within a preliminary time step analysis, the time step size was gradually reduced until convergence of the simulated mixing time curve was achieved. This analysis was done for the GST-2 at 120 rpm due to the highest average velocity gradients and resulted in a time step size of 0.01 s to be sufficient for all vessels. The Courant number was smaller than unity for most mesh cells to ensure numerical stability and convergence. The simulations were run for up to 300 s. Similar to published literature (Spann et al., 2019), a homogenization criterion of 95% was selected to determine mixing times. For a complete representation of the whole vessel, the global mixing indicator $M_{global}$ was chosen which is quantified by the squared deviation of concentrations in the entire fluid domain:

$$\begin{array}{c} M_{global}\left(t\right)=1-\sqrt{\frac{1}{V}\int {\left(\frac{c\left(t\right)}{c_{\infty }}-1\right)}^{2}dV} \end{array}$$ (2)

where V is the vessel volume, $c\left(t\right)$ is the cell concentration over time and $c_{\infty }$ is the volume-average mean concentration. The time to reach 95% homogenization ( $M_{global}=0.95$ ) is the simulated mixing time. For GST-1 (at 60 rpm) experimental mixing time data were performed in the laboratory, whereas for the SUM (at 250 rpm) data were available from the vendor. In both cases, the local tracer concentration at the probe position was taken from the simulation. For the SUM, mixing times for the 99% criterion were available and therefore evaluated.

#### 2.2.4 Mixing time studies—Experimental

Salt spiking experiments could be performed for GST-1 at equal volume and stirrer speed to the CFD simulations. The vessel was filled with desalted water as model fluid. The stirrer speed was set and 30 µL of 1 M KCl (Merck KGaA) solution was manually added with a pipette to the top of the liquid surface. The conductivity of the vessel solution was measured externally: A peristaltic pump (Minipuls 3, Gilson, Middleton, USA) was used to continuously pump the solution through PEEK tubing to an in-line pH/C-900 conductivity monitor (Cytiva, Uppsala, Sweden) at a flow rate of 1 mL/min. Since the influence on the volume of the reactor was assumed to be neglectable (<1%), the outflowing solution was discarded to prevent flow field disturbances. Analogue to the CFD simulations, the mixing time was determined at 95% of the final conductivity. The tubing dead volume was determined and measurements were conducted in triplicates. Mixing time data and position for the SUM were available from the vendor.

#### 2.2.5 CFD reaction modeling of the ADC conjugation reaction

The ordinary differential equations (ODEs) of the kinetic model describing the DAR 2 conjugation reaction scheme were taken from a previous work (Andris et al., 2019). The model consists of two consecutive conjugation steps and a parallel reaction for the payload/drug inactivation:

$$\begin{array}{cc} 1.\;conjugation\;rate=k_{1}\left[mAb\right]\left[Drug\right], & k_{1}=0.797\;{\left(mM*s\right)}^{-1} \end{array}$$ (3)

$$\begin{array}{cc} 2.\;conjugation\;rate=k_{2}\left[mAb_{1Drug}\right]\left[Drug\right], & k_{2}=1.476\;{\left(mM*s\right)}^{-1} \end{array}$$ (4)

$$\begin{array}{cc} Drug\;sink\;rate=k_{3}\left[Drug\right], & k_{3}=0.00155\;s^{-1} \end{array}$$ (5)

In short, the model assumes that mAb and payload react to the mono-conjugate ( $mAb_{1Drug}$ ) and afterwards to the desired bi-conjugate ( $mAb_{2Drug}$ ). The values of the three calibrated rate constants were taken from previous small-scale experiments using the surrogate payload NPM (Andris et al., 2019). In the original model, an initial distribution in % of available cysteines on the mAb is considered which consequently leads to seven ODEs. The percentages of mAbs with two, one and zero activated cysteines were set to 88.59, 8.60% and 2.81%, which was calculated from the final ratio of mAb, mAb_1Drug_ and mAb_2Drug_ in the validation run. Since this assumption does not affect the time-course of the reaction but increases the computational demand, it was thus only adopted in the reaction simulations for the validation for better agreement with the experimental data and neglected in the remaining CFD simulations.

For the CFD reaction models (3D-model), the reaction is considered as homogeneous liquid reaction system. To predict the course of the ADC conjugation reactions in stirred vessels, simultaneously solving the differential equations of momentum, energy, mass and species is required. The ODEs were implemented as volumetric reactions in the species transport equation in FLUENT. This was realized by adding a rate of production *R*_*i*_ and a source term *S*_*i*_ representing the rate of creation to the mass conservation equation which takes the following differential form for the *i*th species:

$$\begin{array}{c} \frac{\partial }{\partial t}\left(\rho Y_{i}\right)+\nabla ∙\left(\rho \vec{v}Y_{i}\right)=-\nabla \vec{J_{i}}+R_{i}+S_{i} \end{array}$$ (6)

where ρ is the liquid density, ν is the fluid viscosity, *Y*_*i*_ is the species mass fraction and $\vec{J_{i}}$ is the diffusion flux of species *i*. Constant density was assumed for all species and the increase in volume was neglected due to the rather small volume of added payload of 1.67% (v/v) with respect to the total volume. The finite-rate model was chosen to calculate the production term. This approach computes the chemical reaction rate of each species directly with neglecting turbulence-chemistry interaction. Backward reactions were set to zero and the liquid temperature was assumed isothermal. For initialization, the entire fluid domain was homogenously patched with the desired mAb concentration. The payload addition was performed sub-surface. Addition positions can be found in the Supplementary S3. Instant addition was implemented by initializing a spherical domain with a payload concentration of 10 mM. For addition over time a source term in the same region was implemented, which generated a constant amount of payload in each time step for the feeding times. A time step size of 0.01 s was also used for these studies. This was determined by initializing the entire volume for GST-2 as perfectly-mixed (as assumed in the 0D-model) and lowering the time step size until the kinetics converged against the predictions of the 0D-model. The transient reaction simulations were performed with similar settings as the mixing time studies and were run for 300 s since all mAb is conjugated during this period.

#### 2.2.6 Validation of CFD reaction modelling

A validation run for the CFD reaction modeling was performed in the GST-1 vessel using 300 mL mAb solution with 1.5 mg/mL (ADC1) and 5x molar payload excess. The stirrer speed was set to 60 rpm and a surrogate payload dissolved in DMSO was added over 1 min with a syringe pump (Nemesys S). Time samples were quenched with NAC and the conjugation kinetic of the intact ADC species was determined with a non-reducing RP-UHPLC method. The same protocol, system and column as described in (Andris et al., 2019) was used.

#### 2.2.7 Vessel comparison and parameter study

Using the 3D-models, the kinetics in three mixing vessels were compared. Furthermore, a parameter study was conducted, exemplarily for GST-2, that covered the variation of three process parameters in typical ranges. The investigated parameters are summarized in Table 2. Only one parameter was varied at a time while the other parameters were kept constant at the standard condition of 60 rpm, 5 mg/mL mAb concentration, 5x molar payload excess and 60 s addition time.

**TABLE 2:** Investigated process parameters within the parameter study conducted for the GST-2. Standard conditions were 60 rpm, 60 s addition time, 5 mg/mL mAb and 5x molar payload excess.

Parameter	Range
Stirrer speed	60, 80 and 120 rpm
Payload addition time	0 (Batch), 60 and 300 s
c_mAb_	5 and 10 mg/mL

Analogously, the original kinetic model was expanded with a fed-batch term assuming ideal-mixing. This resulted in a classical 0D-model which serves as a reference for the effect of vessel scale and process parameter when comparing the outcome of 0D- and 3D-model. This deviation between both simulations was quantified using the absolute difference in the DAR value over time:

$$\begin{array}{c} ∆DAR\left(t\right)=DAR_{0D}\left(t\right)-DAR_{3D}\left(t\right) \end{array}$$ (7)

whereas the DAR values were calculated with:

$$\begin{array}{c} DAR\left(t\right)=\frac{c_{mAb,1Drug}\left(t\right)+{2*c}_{mAb,2Drug}\left(t\right)}{c_{mAb,0}}. \end{array}$$ (8)

In case of the 3D-model, the concentrations of the conjugated species were obtained from the volume-averaged species concentrations at each time step. The 0D-model was simulated in MATLAB R2020a and the differential equations were solved using the *ode45* solver.

#### 2.2.8 Time-scale analysis

As an alternative approach to fully modeling the dynamic reaction, one can compare the time-scales of reaction and mixing to receive an expectation regarding the predominating mechanism to be considered. An inhomogeneity of reactant concentration in large-scale reactors operated in fed-batch may be caused by weak distribution of the added reactants. This characteristic time can be quantified with the mixing time (Nadal-Rey et al., 2021). For chemical reactions one can calculate the characteristic time $\tau_{R}$ for a bimolecular reaction according to (Vicum et al., 2004):

$$\begin{array}{c} \tau_{R}=\frac{1}{k_{i}\left(\tilde{c_{1}}+\tilde{c_{2}}\right)}, \end{array}$$ (9)

where $k_{i}$ is the kinetic rate of the *i*th reaction and ${\tilde{c}}_{j}$ is the local concentration of the *j*th species. If the characteristic reaction time is significantly larger than the mixing time, the reaction can be considered as ideal-mixed, while for larger mixing times in comparison to the reaction time the process becomes mixing-sensitive.

## 3 Results and discussion

The first part of this chapter deals with the experimentally determined conjugation kinetics and the comparability between small- and lab-scale conjugation kinetic. In the second part, the CFD results for the three studied vessels are analyzed involving typical scaling parameters such as P/V, resulting flow fields and mixing times. The chosen process parameters at which the vessels were compared can be found in Table 1. Finally, the CFD-simulated ADC conjugation kinetics are compared among the vessels and the influence of process parameters on the reactions is studied for GST-2.

### 3.1 Experimental conjugation kinetic studies

#### 3.1.1 Small-scale conjugation kinetic studies

**FIGURE 1:**
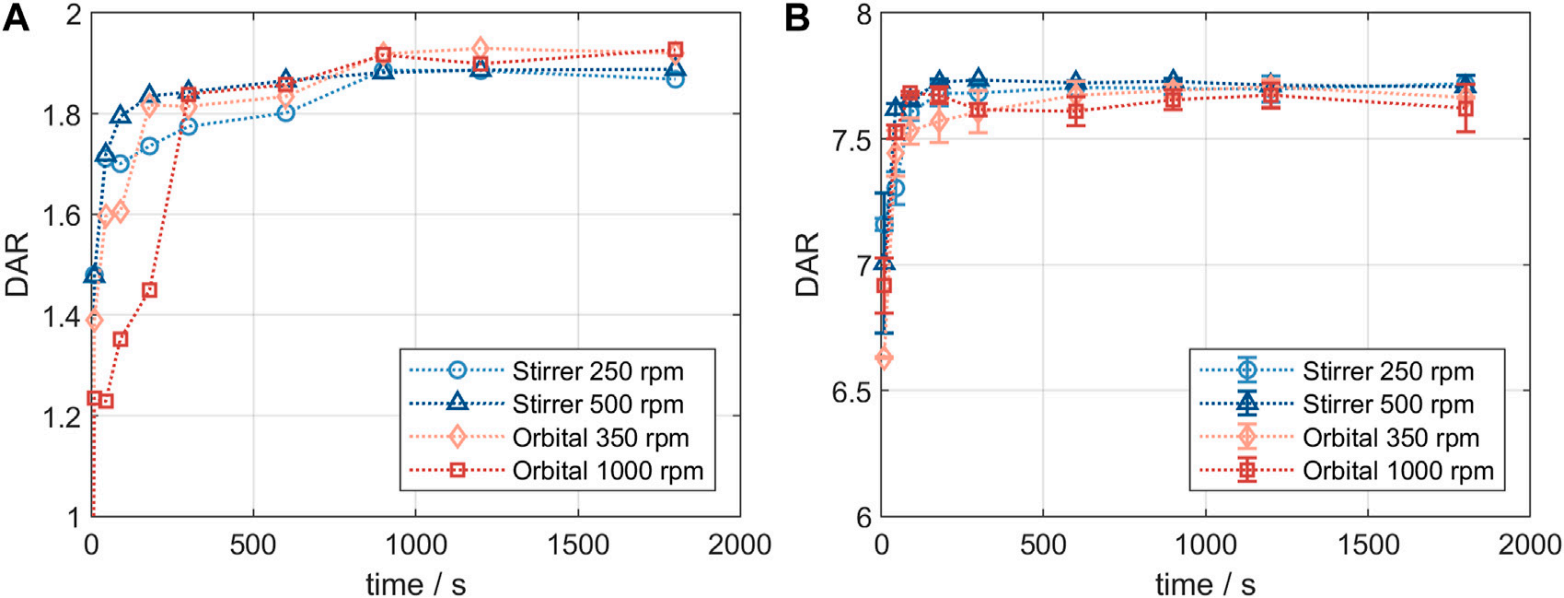
Comparison of the RP-UHPLC determined DAR conjugation kinetics performed in small-scale reaction tubes with different mixing systems and speeds. **(A)** ADC1 at a concentration of 10 mg/mL conjugated with 5x molar payload excess and **(B)** ADC2 at a concentration of 1.5 mg/mL conjugated with 11x molar payload excess. Runs were performed in duplicates and error bars represent the standard deviations.

The resulting DAR kinetics from the RP-UHPLC analysis are shown in Figure 1A for the DAR 2 species. The DAR increases rapidly for all reactions and the mAb is entirely conjugated after 900 s. Notably, the curves deviate during the initial phase of the conjugation reaction with the orbital shaker at 1,000 rpm having the largest offset from the ideal conjugation kinetic. This might be caused by an experimental artefact, but also demonstrate the requirement for proper mixing which cannot be ensured using the orbital shaker. Since conjugation reactions were performed over 1 h and the curves deviate only during the initial reaction period, the final DAR values and the drug load profile (data not shown) were not affected by the mixing type. The achieved average final DAR of all runs is 1.90 which is lower than the theoretical DAR of 2. This can be caused by the previously described pre-inactivation of cysteines (Andris et al., 2019) that originate from reformed disulfide bridges from reactive thiols formed during the reduction and re-oxidation step (Coumans et al., 2020). The averaged kinetics for the DAR 8 species are shown in Figure 1B). The error bars represent the standard deviation of each duplicate. Similarly, the DAR curve increases rapidly while final DAR values are reached at approx. 300 s. All kinetics show no considerable deviation between the mixing types and final DAR values range between 7.5 and 7.8. Overall, the effect of the mixing on the final DAR for both molecules was rather small if the reaction was run for 1 h. In summary, the conjugation kinetic study demonstrated that internal stirring in reaction tubes may be favored. Using the orbital shaker only results in acceptable kinetics when initial mixing is conducted properly.

#### 3.1.2 Conjugation kinetic comparison of reaction tubes and lab-scale stirred vessel

**FIGURE 2:**
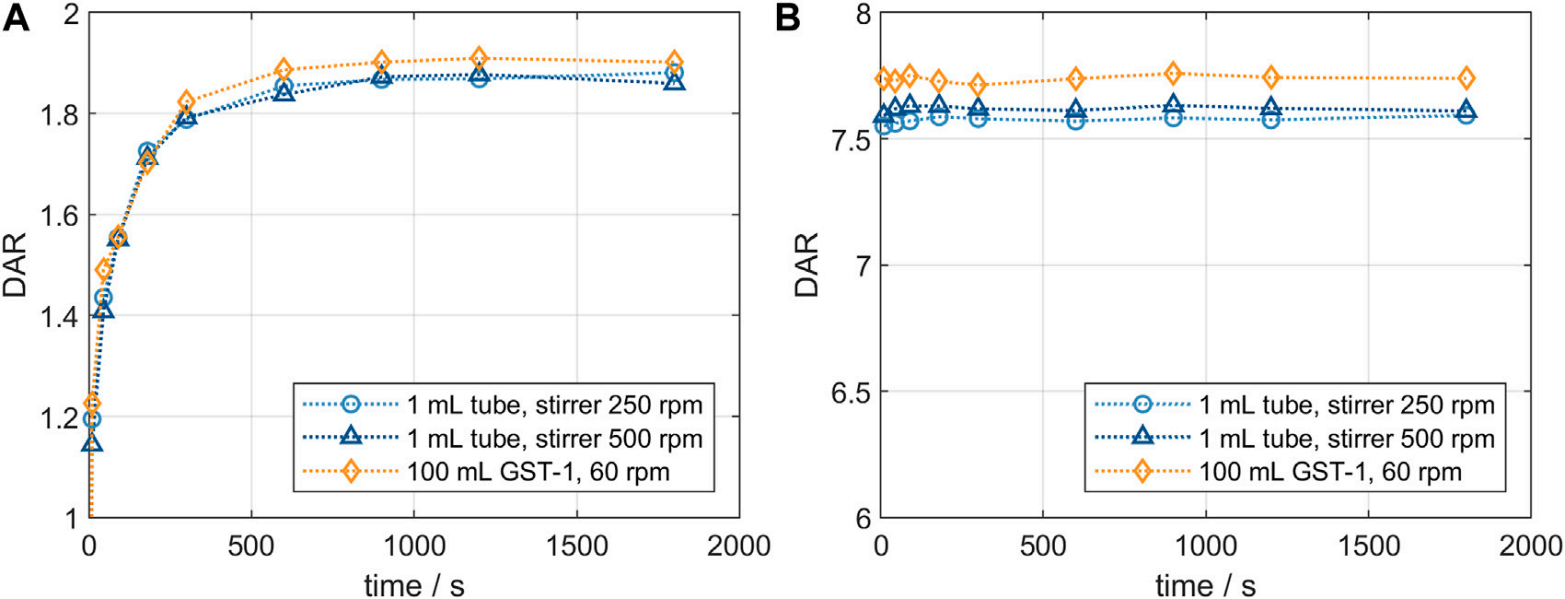
Comparison of the small- and lab-scale DAR conjugation kinetics determined by RP-UHPLC. **(A)** Kinetics for ADC1 at 5 mg/mL and 5x molar payload excess and **(B)** kinetics for ADC2 at 20 mg/mL and 11x molar payload excess.

Product quality and DAR of an ADC are usually tested after the conjugation reaction is complete. It was of interest to study changes in conjugation state over the course of the reaction and to compare the small-scale reaction tubes with a larger reactor set-up. Figure 2 shows the comparison of the DAR-course in reaction tube vs lab-scale stirred tank (GST-1) for both the site-specific conjugation to inserted cysteines (left panel) and the stochastic conjugation to reduced interchain disulfide bonds (right panel). For both ADCs the kinetics follow the same course at the two scales. In both cases slightly higher DAR values are achieved in the lab-scale vessel. However, the observed difference is within assay variability. Furthermore, no considerable difference in the drug load profiles was present (data not shown). These experiments demonstrate that the internally stirred reaction tube shows good comparability to the lab-scale vessels for the conjugation chemistries studied.

### 3.2 CFD simulations for large-scale vessels

#### 3.2.1 Large-scale vessel characterization using steady-state and mixing time simulations

**FIGURE 3:**
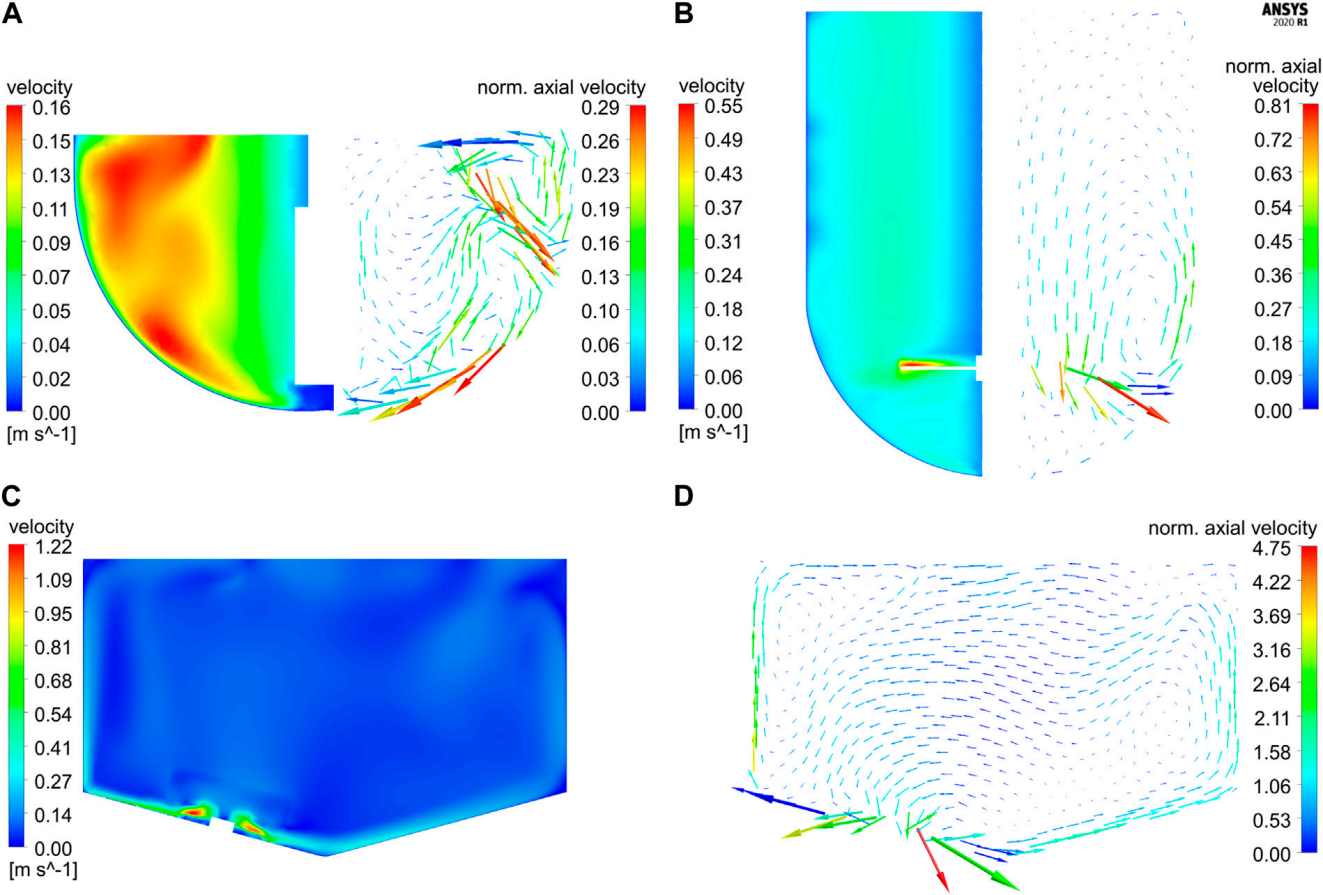
Contour plots of the velocity magnitude vs projected velocity vector plots colored according to the axial velocity magnitude (normalized to the respective average velocity magnitude in each reactor) on vertical cut planes for **(A)** GST-1 (60 rpm), **(B)** GST-2 (60 rpm) and **(C–D)** SUM (400 rpm). Due to the symmetrical design of GST-1 and -2, half of the plots are shown side-by-side. Please note that the vector length corresponds to the velocity magnitude in each reactor but was scaled individually for each plot and, therefore, is not comparable among the reactors.

Simulations for both glass vessels GST-1 and GST-2 result in comparable P/V values of 3.81 and 2.44 W/m³, respectively. A tenfold higher P/V value of 23.71 W/m³ is reached in the SUM due to its higher stirrer speed. Figure 3 shows the resulting plots for the velocity magnitude and projected velocity vectors of the steady-state solution for the three studied vessels. The vectors are colored by the magnitude of the axial velocity normalized to the average velocity magnitude in each vessel, in order to examine regions which contribute to the axial transport in the vessel. The comparison of the contour plots demonstrates that in the GST-1 overall a larger part of the bulk has higher relative velocities than the other two reactors. In the GST-2, high velocities were found near the stirrer and medium velocities in the remaining bulk. For both vessels, velocities close to the shaft are lower. In the SUM, high velocities occur near the impeller blades. In contrast to the glass vessels, the majority of the bulk appears to have lower velocity magnitudes compared to the impeller tip speed which is indicated by a larger amount of (light) blue areas in the contour plots. This is due to the impeller discharge towards the bottom of the vessel and the smaller impeller diameter in relation to the vessel diameter. Notably, the average velocity magnitudes of all three reactors are in a similar range. When comparing the vector plots it becomes obvious that the flow direction in the vessels differ strongly. For the two glass reactors, the flow was found to be mainly rotational, but in the GST-1 a larger region appears to contribute more to the axial transport, which is indicates by more vectors having higher axial velocities. For the GST-2, only flow close to the stirrer region contributes to axial transport, whereas the volume above the impeller is mainly rotational with very low axial velocities. This is due to the lack of baffles and the relatively low stirrer speed in comparison to the reactor volume. In contrast, the vectors for the SUM indicate much higher axial (and also radial) velocities, especially in the area close to the sides of the vessel where flow is directed upwards. Moreover, the average axial velocity is approx. Twofold higher than in the other two vessels. The directions of the vectors emphasize that the eccentric position of the impeller also produces a more chaotic and asymmetric flow field with higher gradients in axial/radial direction compared to the glass reactors potentially leading to improved mixing.

The mixing performance of the three reactors was compared based on the CFD mixing time studies. The fastest homogenization is achieved in the GST-1 with a predicted (global) mixing time of 9.4 s due to small volume being relatively well mixed which agrees with the high velocities with axial transport in the bulk. In contrast, the mixing time in the GST-2 is much slower with 32.2 s. Especially, the final homogenization close to 95% in this vessel is observed to be relatively slow. This is caused by the strong rotational flow and relatively low axial and radial transport which coincides with the findings from the vector plots. The mixing in the SUM is remarkably faster with a mixing time of 17.6 s, although the liquid volume is similar to GST-2. This is caused by the higher stirrer speed and an intensified mixing efficiency due to the eccentric impeller design leading to higher axial/radial transport. The simulated mixing curves are depicted in the Supplementary Figure S6. It is worth mentioning, that using the relation between the reaction times (ranging between 300—900 s) and the large-scale mixing times (ca. 10–30 s), one can expect only minor mixing dependency on the reaction at this point.

#### 3.2.2 CFD model validation

**FIGURE 4:**
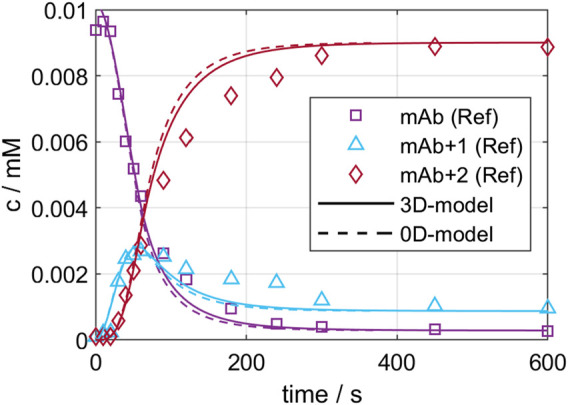
Comparison of 0D- and 3D-model predictions of the ADC conjugation kinetic in GST-1 with the experimentally determined kinetic for the first 600 s.

On the one hand, the developed CFD models were validated by comparing local mixing times. The experimental and simulated mixing times are 9.1 and 8.1 s for GST-1, and 49.0 and 45.2 s for the SUM. This results in an error of 10.0% and 7.8% for the GST-1 and SUM, respectively, which indicates a potential mismatch between experiments and CFD simulation. This can originate from multiple root causes, such as model simplification through the isotropic flow assumption by the applied RANS model, the MRF technique, frozen flow field or inequality between real and simulated measurement position. Similar errors in the range of 10% were reported in literature (Scully et al., 2020), (Martinetz et al., 2021) which led to the assumption that the observed deviation are in an acceptable range for the purpose of this study. On the other hand, the validity of the kinetic models to predict large-scale were investigated with a conjugation run in the GST-1, as described in chapter 2.2.6. Figure 4 presents the predicted kinetics for the three ADC species of both models compared to the reference data from the RP-UPHLC. Both models show a very similar course for all species which is also shown in similar *R* ^2^ values of 0.979 and 0.985 for the 0D- and the 3D-model, respectively. Compared to the reference data both models have an offset between 200—500 s while converging simultaneously to equal species concentrations. These results emphasize the agreement of both model types at this particular scale.

#### 3.2.3 3D kinetic modeling of large-scale vessels

**FIGURE 5:**
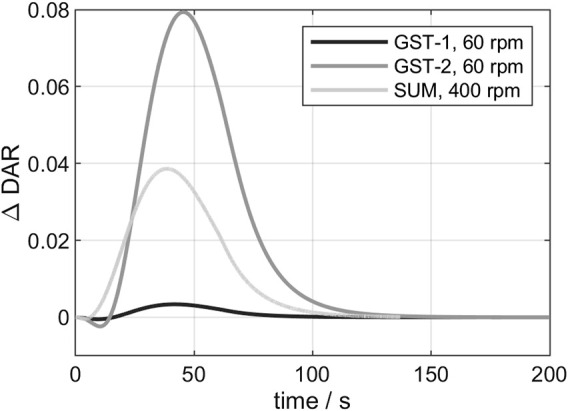
Comparison of the ∆DAR within the first 200 s for all three studied vessels. Payload addition was simulated over the course of 60 s.

In the following, the 3D conjugation kinetics of the three vessels (see chapter 2.2.7) are compared. For the examination of the deviation between 3D- and 0D-model, the $∆DAR\left(t\right)$ is shown for the three vessels in Figure 5. According to this graph, two zones can be distinguished: In the first zone, which is in the beginning of the reaction, the ∆DAR curves increase exponentially and reach a maximum value depending on the vessel. Hereby, GST-1 has the smallest deviation (0.0035) and GST-2 the largest deviation (0.08). In the second zone, the three curves converge to $∆DAR\left(t\right)=0$ after around 80—130 s. The initial increase in the ∆DAR is due to the local availability of added payload in the feed region. The resulting mass transfer limitation leads to actual lower kinetic rates in the remaining bulk which cannot be captured by the 0D-model. The magnitude of this increase (GST-2 > SUM > GST-1) was found to qualitatively agree with the order of the simulated mixing times.

**FIGURE 6:**
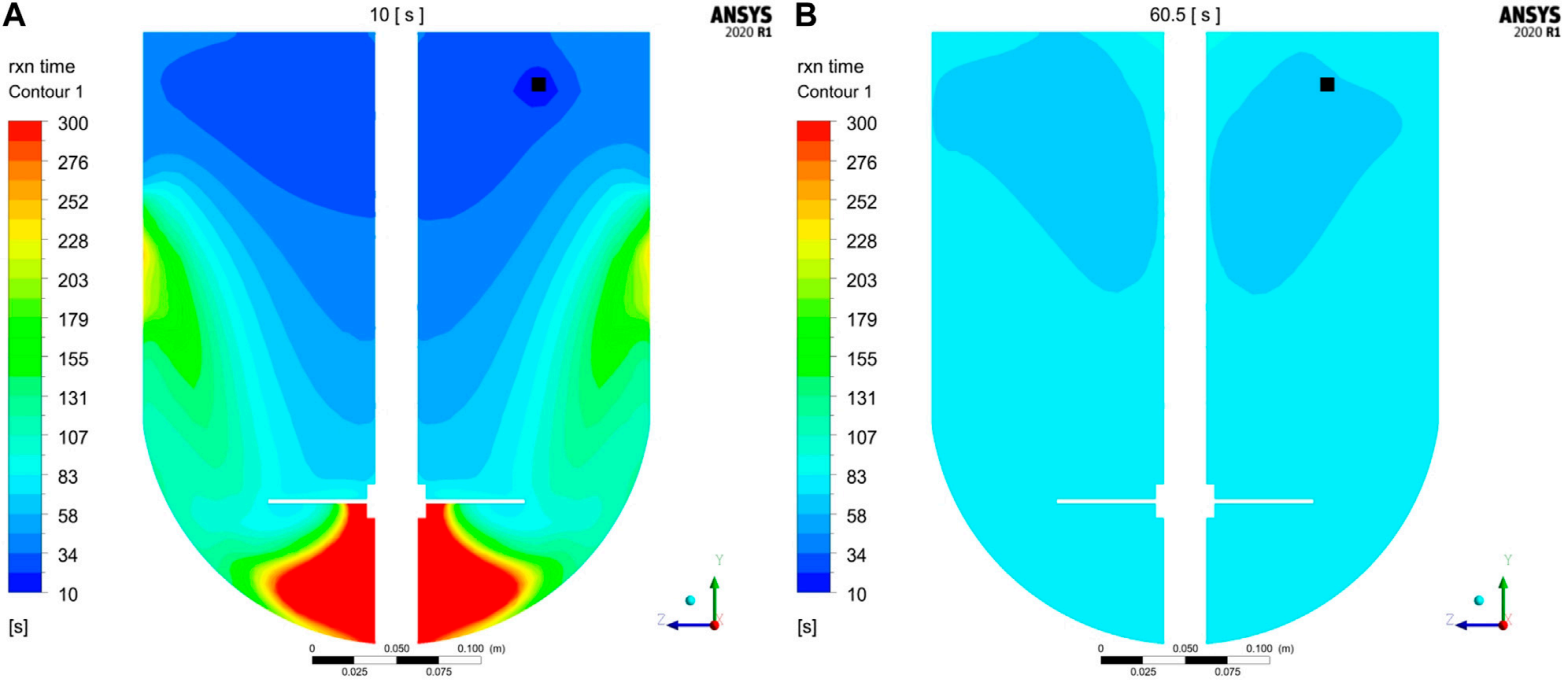
Contour plots of reaction time-scales (1. Conjugation step) at vertical cut planes compared for **(A)** 10 s and **(B)** 60.5 s exemplary calculated for GST-2. Feeding position is colored in black.

Additional insights were gained by studying the reaction time-scales at different process times. Local reaction time-scales were exemplarily computed for the first conjugation step at 10 and 60.5 s (immediately after addition is finished at 60 s) according to Eq. (9). The resulting contour plots of the reaction times are shown exemplarily for GST-2 in Figure 6. It is noticed that at 10 s the reaction times are lower in the upper part of the vessel. This is equivalent to fast reaction rates, which is due to the freshly added payload in this region. In contrast, the low mass transfer to the region underneath the impeller are found to reduce the reaction rate in this region which is indicated by higher reaction times (green to red areas). This agrees with the observation of the low axial transport downwards to the lower part of the vessel (see Figure 3B). In contrast, reaction times are more homogenously distributed after payload feeding is finished (60.5 s) due to a higher degree of homogenization. In summary, this analysis reveals that in the initial phase, reaction times are slightly lower but in a similar magnitude compared to the mixing times which is 32.2 s. Thus, it can be concluded that the speed of homogenization of freshly added payload is responsible for a slight reduction of the conjugation rate in the beginning of the process. However, the calculated maximum ∆DAR values are in a rather irrelevant industrial range, especially, since actual process times are greater than 100 s. The same estimation can be made when calculating the average reaction time by using the mean concentrations of mAb and payload which highlights the benefit of time-scale analysis. Moreover, it should be mentioned that, since the applied conjugation reaction is a consecutive reaction, the deviations in the kinetic of all species are only temporally affected by the vessel mixing and have no large influence on the final DAR value. In literature, the similarity between a CFD (3D) and an ideal-mixing model (0D) have also been shown by Spann et al. (Spann et al., 2019) for a fermentation biokinetic model. In this study, the authors presume that the observed local pH changes may not affect the biokinetic and those small differences actually originate from numerical errors in the CFD simulation. In another publication (Nadal-Rey et al., 2021), the authors emphasize the advantage of conducting a time-scale analysis in order to determine possible effects of mixing gradients on reactions. It is also contrasted that this analysis does not provide information about the possible effects on relevant CQAs, hence not replacing experimental or *in silico* studies.

#### 3.2.4 Modeling the influence of process parameters

**FIGURE 7:**
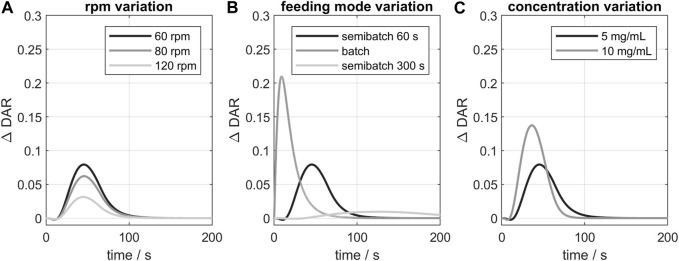
Influence of varying process parameters on the ∆DAR of 3D- and 0D-model exemplarily for GST-2: variation of **(A)** stirrer speed, **(B)** feeding mode, **(C)** mAb concentration. The black curve is the reference condition (60 rpm, 60s feeding time, 5 mg/mL + 5x payload excess).

Additionally, the influence of varying process parameter on the kinetic was studied exemplarily for GST-2. The resulting curves of the ∆DAR are shown in Figure 7. A stepwise increase of the stirrer speed from 60 to 120 rpm leads to a decrease of the model deviation which can be attributed to enhanced mixing performance caused by greater velocity gradients and turbulence. However, increasing the stirrer speeds appears to have a rather small effect on improving the mixing performance and, thus, reducing the ∆DAR value. A larger influence is observed for the investigated feeding modes (see Table 2): The batch addition causes the ∆DAR to increase rapidly to around 0.2 compared to semibatch mode. This behavior can be related to a higher amount payload locally available at the same time producing more inhomogeneities which cannot be captured in the 0D-model. Semibatch feeding led to lower maximum ∆DAR . For 300 s feeding the models deviate only marginally which shows that an increase in feeding time is likely to minimize mixing effects or other phenomena due to locally high payload concentrations. Doubling the mAb concentration also resulted in a short increase of the ∆DAR . Here, the mixing effect is more pronounced due to faster initial reaction rates. As illustrated by the studied parameters, any changes of the process parameters would only influence the time of completion of the reaction but would reach the same endpoint.

In conclusion, the 3D-model indicated deviations from the ideal conjugation kinetic, especially when all payload is added at once (batch mode) or for higher reactant concentrations. For the studied vessel (GST-2), the stirrer speed had only little influence on the course of the kinetic. Other parameters, like feeding position, were also studied but showed even smaller deviations and are therefore not presented here. Overall, the influence of geometry and process parameters were generally small in the case of the studied consecutive two-step conjugation reaction. This is due to the naturally selective conjugation to the targeted sites which has been reported to simplify process development and scale-up (Hutchinson et al., 2018). Furthermore, mixing in the studied vessels is adequate due to relatively the slow (bio-chemical) kinetic reaction in contrast to other typical faster chemical reactions. A complete validation of the predicted species time-course would also be necessary. In our case, the results of the CFD kinetic study gave a comprehensive overview of possible parameter influence on the course of the ADC conjugation kinetic. For stochastic conjugation chemistries, the parameter effects may be relevant and the CFD model might be more advantageous. Moreover, the CFD model could be used to predict other effects like shear rate-depended mAb fragmentation or aggregation which was not observed in this study and would require a additional model to be incorporated.

## 4 Conclusion

This work considered different aspects for a better understanding how scale-up and process parameters affect the ADC conjugation reaction for two model ADCs. First, experimental kinetic studies in reaction tubes dealt with the optimization of mixing by using different mixing types. We could show that reaction tubes that are internally mixed using a magnetic stir bar produce consistent conjugation kinetics which are comparable to kinetics in glass reactors. Secondly, different types of CFD simulations were performed for three commonly used vessels in ADC manufacturing. Using steady-state simulations and mixing time studies we could characterize the vessels’ mixing performance and were able to describe local mixing effects. We further implemented a DAR 2 conjugation kinetic model in the CFD models leading to a full 3D-model. By using the classical ideal-mixing model (0D) as benchmark we showed that the relation of achieved mixing times and chemical reaction rates governs the implications obtained during scaling. Current ADC conjugation reactions are, however, in a range where mixing performance in commercially available vessels is adequate for the fast conjugation kinetic. This indicated that the ratio of mixing time and chemical reaction kinetic is a reliable indicator to be considered during scaling for this reaction type. The often-applied P/V value did not show to correspond well with the observed deviations. Furthermore, we studied variations in process parameters (stirrer speed, feeding mode and concentration variation). We found that the parameters affected the conjugation kinetic only little within the first 100 s of the reaction and final DAR values remained constant. This can be attributed to the highly selective conjugation chemistry and the consecutive nature of the reaction. A time-scale analysis demonstrated that conjugation rate inhomogeneities occur in the feed region and only during the addition phase. In the case of the DAR 2 conjugation reaction, the additional insight from the 3D-model were rather not industrially relevant. Therefore, the 0D-reactor models can be applied for predicting large-scale conjugation kinetics and to be used in a digital twin framework. As this study was successfully conducted for a DAR 2 conjugation reaction it has the potential to be adopted to other (conjugation) reactions.

## Data Availability

The raw data supporting the conclusion of this article will be made available by the authors, without undue reservation.
