# Corrigendum: Kinetic studies and CFD-based reaction modeling for insights into the scalability of ADC conjugation reactions

**DOI:** 10.3389/fbioe.2023.1229416

**Published:** 2023-06-09

**Authors:** Jan Tobias Weggen, Janik Seidel, Ryan Bean, Michaela Wendeler, Jürgen Hubbuch

**Affiliations:** ^1^ Institute of Process Engineering in Life Sciences, Section IV: Biomolecular Separation Engineering, Karlsruhe Institute of Technology (KIT), Karlsruhe, Germany; ^2^ Purification Process Sciences, BioPharmaceuticals Development, R&D, AstraZeneca, Gaithersburg, MD, United States

**Keywords:** antibody-drug conjugate (ADC), conjugation reaction, computational fluid dynamics (CFD), mixing, scale-up, single-use, kinetic modeling

In the published article, there was an error in **Affiliation** [2]. Instead of “Purification Process Sciences, BioPharmaceuticals Development, Gaithersburg, MD, United States” it should be “Purification Process Sciences, BioPharmaceuticals Development, R&D, AstraZeneca, Gaithersburg, MD, United States”.

The authors apologize for this error and state that this does not change the scientific conclusions of the article in any way. The original article has been updated.

