# Supplementary material for: Kinetic studies and CFD-based reaction modeling for insights into the scalability of ADC conjugation reactions
Source: Front Bioeng Biotechnol. 2023 Apr 3;11:1123842. doi: 10.3389/fbioe.2023.1123842 (PMC10111256; doi:10.3389/fbioe.2023.1123842)
Supplement: Supplementary file 1 [file DataSheet1.DOCX]

# Supplementary Material

## S1 Experimental mixing study for DAR8
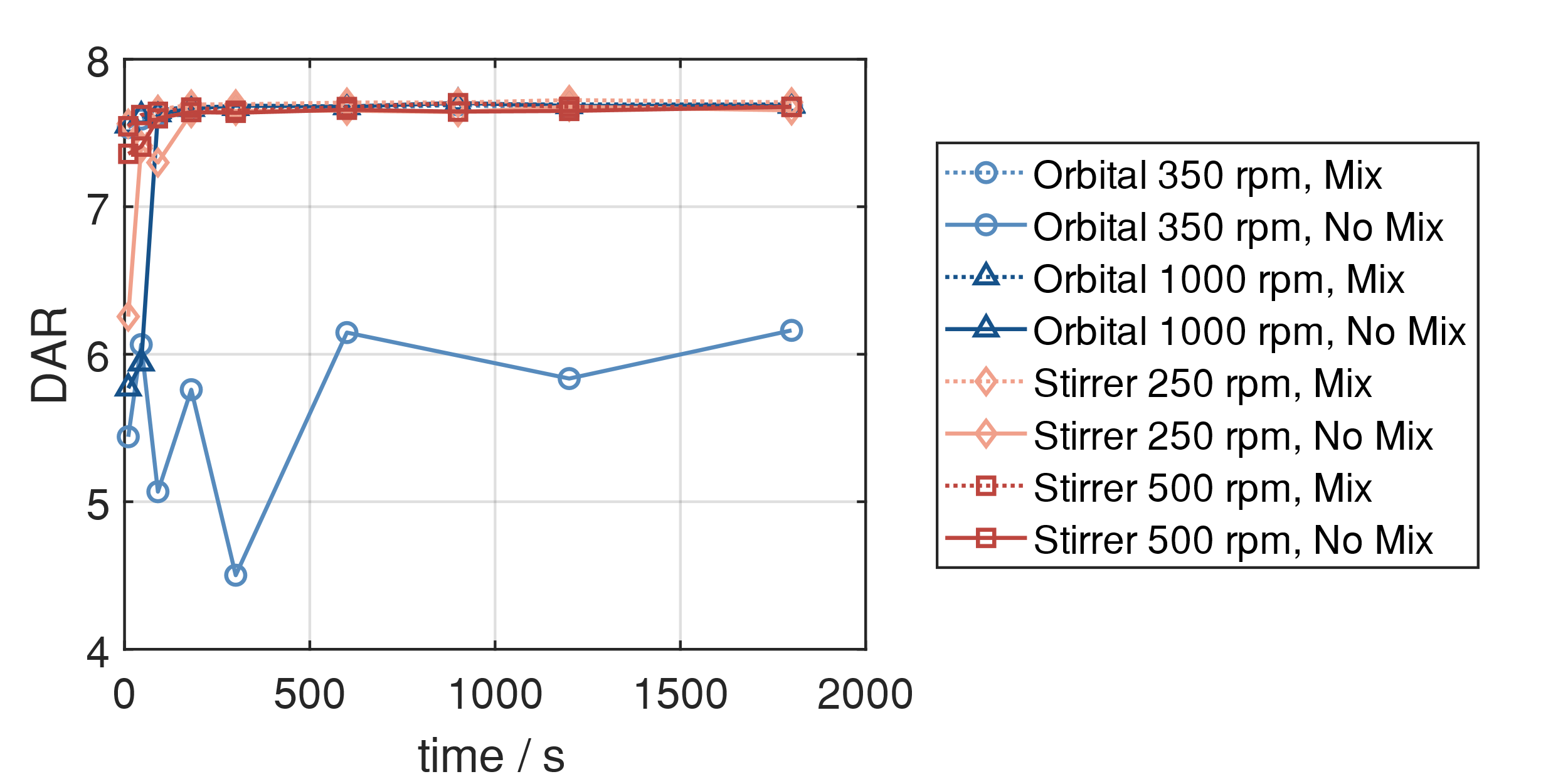


Figure 1: DAR kinetics for different mixing modes with and without initial payload mixing performed. Reaction conditions were 1.5 g/L mAb and 14x molar payload excess.

## S2 CAD Geometries

Table 1: Vessel, stirrer and shaft dimensions of the CAD models.

|  | **GST-1** | **GST-2** | **SUM** |
| --- | --- | --- | --- |
| **Inner diameter vessel / mm** | 108 | 300 | 384 |
| **Vessel bottom type** | Curved spherical | Curved spherical | Cone |
| **Height of the curved bottom / mm** | 45 | 130 | 52.7 |
| **Impeller diameter / mm** | 81 | 152 | 65 |
| **Impeller width / mm** | 35 | 20 | 11 |
| **Impeller angle** | 0° | 45° | 15° |
| **Impeller clearance / mm** | 10 | 85 | 4.8 |
| **Shaft diameter / mm** | 7.2 | 25.4 | - |

## S3 Generated meshes

Table 2: Mesh metrics for the three final meshes and feeding positions in this study.

| Reactor | 300 mL GST-1 | 22 L GST-2 | 25 L SUM |
| --- | --- | --- | --- |
| Stirrer speed | 60 rpm | 120 rpm | 400 rpm |
| Mesh count | 206123 | 582731 | 940652 |
| Ortho min (average) | 0.21 (0.94) | 0.2 (0.96) | 0.20 (0.98) |
| Skew max | 0.79 | 0.8 | 0.80 |
| Poly-Hexcore  meshes | 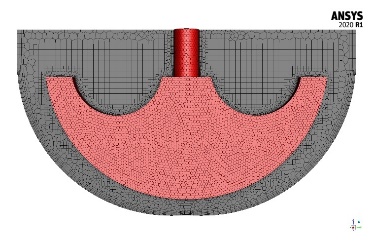 | 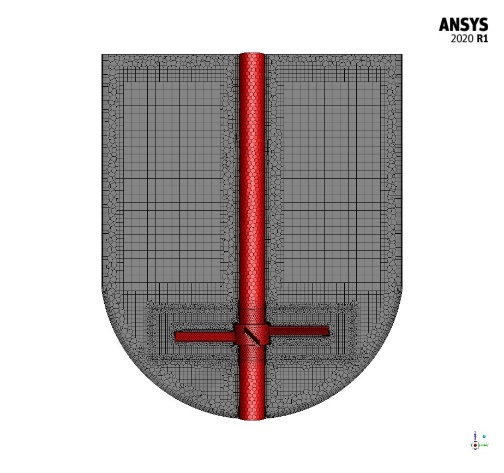 | 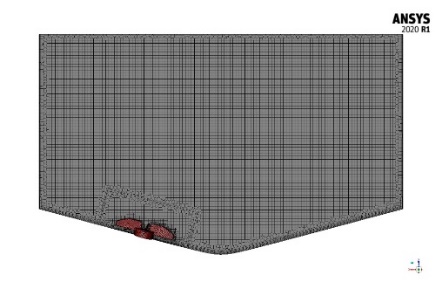 |
| Tracer/ Payload addition positions | 2.5 mm below surface and 2 cm distance to wall | 1.5 cm below surface centered in between wall and tank middle axis | 2.5 cm below surface and 3 cm distance to wall |

## S4 Global mesh study

|  | **Volume-averaged velocity magnitude** | **Turbulence energy dissipation (**$\boldsymbol{\epsilon}$**) /Turbulence kinetic energy (**$\boldsymbol{k}$**)** |
| --- | --- | --- |
| 300 mL GST-1 (60 rpm) |  |  |
| 22 L GST-2 (120 rpm) |  |  |
| 25 L SUM (400 rpm) |  |  |

Figure 2: Mesh independency test for the three vessels (at highest rpm) with regards to volume-averaged velocity magnitude and turbulent energy dissipation and turbulence kinetic energy.

## S5 Convergence monitors for steady-state simulations using the final meshes


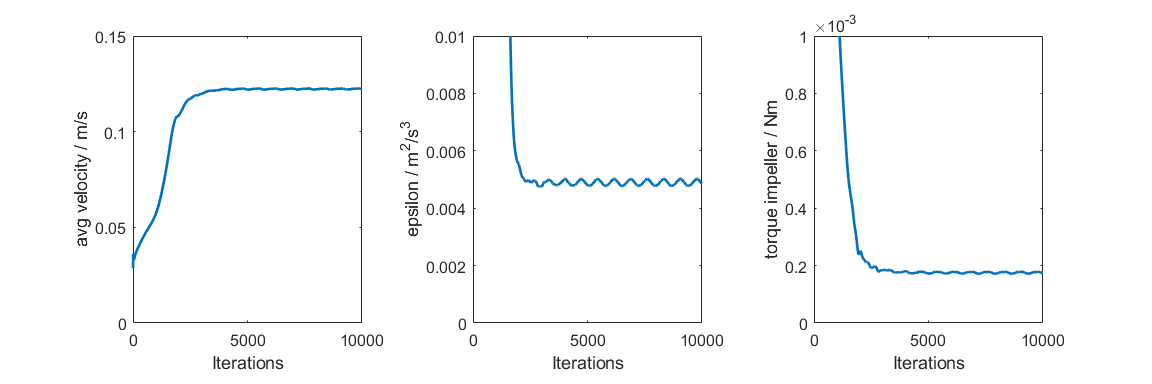

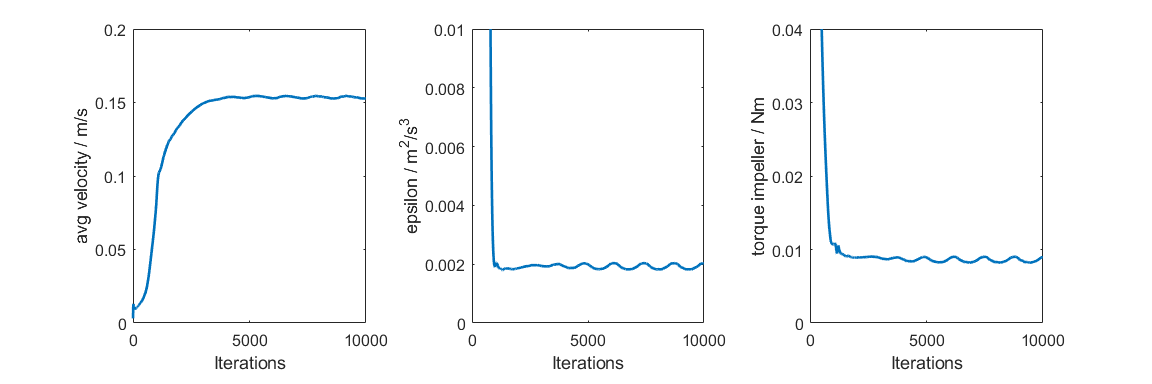

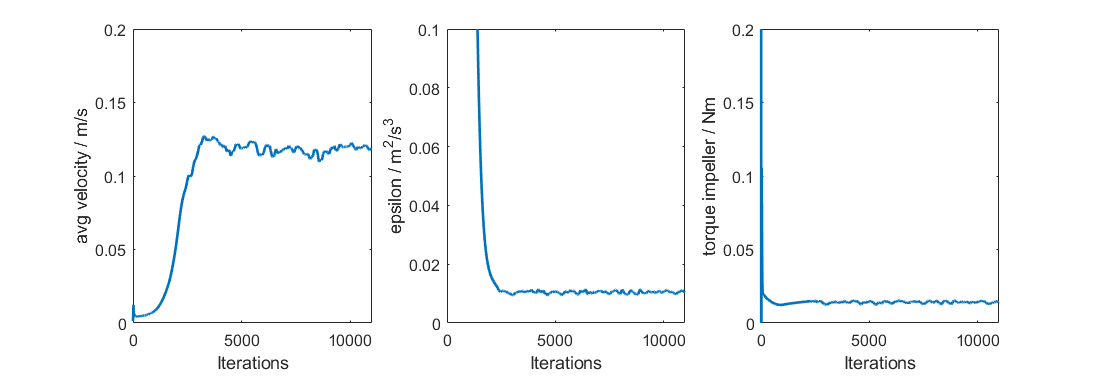


Figure 3: Monitored values of averaged velocity magnitude, turbulent energy dissipation and impeller torque for the three vessels over the iterations during the steady-state simulations. Top: GST-1 (60 rpm), middle: GST-2 (60 rpm) and bottom: SUM (400 rpm).

## S6 Simulated mixing times


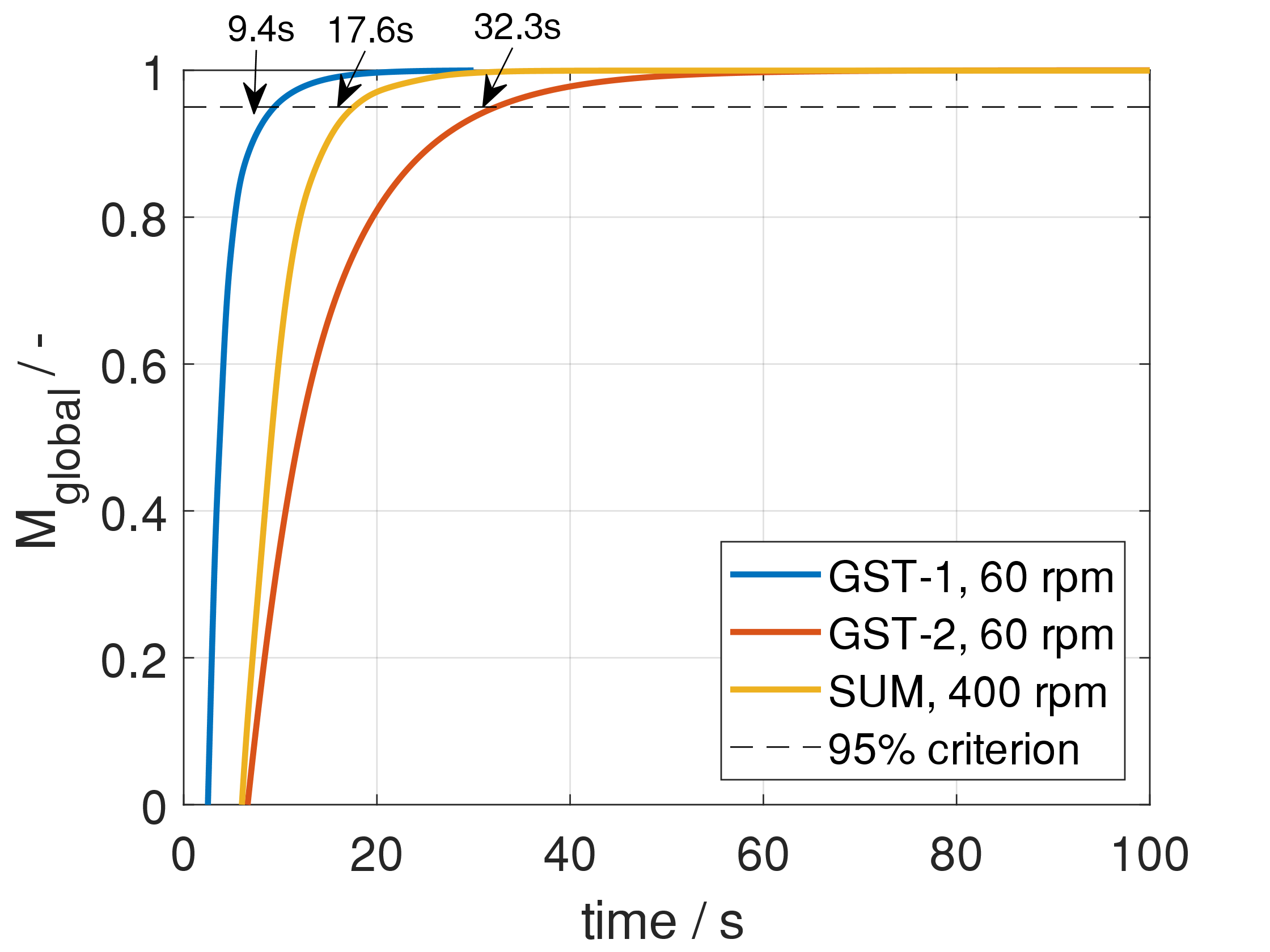


Figure 4: Simulated global mixing curve for all three studied vessels with characteristic mixing times determined for $\boldsymbol{M}_{\boldsymbol{global}}\boldsymbol{=0.95}$.
